# Vinorelbine Plus Platinum in Patients with Metastatic Triple Negative Breast Cancer and Prior Anthracycline and Taxane Treatment

**DOI:** 10.1097/MD.0000000000001928

**Published:** 2015-10-30

**Authors:** Meiying li, Ying Fan, Qing Li, Pin Zhang, Peng Yuan, Fei Ma, Jiayu Wang, Yang Luo, Ruigang Cai, Shanshan Chen, Qiao Li, Binghe Xu

**Affiliations:** From the Department of Medical Oncology, Cancer Institute and Hospital, Chinese Academy of Medical Sciences and Peking Union Medical College, Beijing, China.

## Abstract

Currently, there is no preferred standard chemotherapy regimen available for patients with metastatic triple negative breast cancer (mTNBC) and no cohort studies on the efficacy of vinorelbine plus platinum (NP) regimen in patients with mTNBC who failed to anthracyclines and/or taxanes have been reported. We present the single-center, retrospective experience of NP regimen in a total of 41 patients with mTNBC.

All patients were treated with NP regimen, main combination used was vinorelbine-cisplatin in 34 patients (82.9%).

The median follow-up was 36.8 months. Objective response rate was 34.1% (n = 14) in the whole study group. Three patients experienced complete response (7.3%), 11 patients acquired partial response (26.8%), stable disease was observed in 14 patients (34.1%), and 10 patients (24.4%) had progressive disease. Response evaluation was not applicable in 3 patients who received the treatment of NP regimen after surgical removal of the metastatic lesions. The median overall survival and progression-free survival were 18.9 months (95% confidence interval, 15.6–22.1 months) and 6.7 months (95% confidence interval, 2.9–10.5 months), respectively. The main adverse events were grade 3/4 neutropenia (n = 20, 48.8%) and grade 1/2 gastrointestinal toxicity (n = 20, 48.8%).

NP regimen is active and tolerable in patients with mTNBC pretreated with anthracyclines and/or taxanes. Therefore, among other chemotherapy regimens, NP combination may provide a rational treatment option for this patient subset.

## INTRODUCTION

Triple negative breast cancer (TNBC) accounts for approximately 12% to 17% of all breast cancer and is characterized by absent or minimal expression of hormone receptor [estrogen receptor and progesterone receptor (PR)] and human epidermal growth factor receptor 2.^1^ TNBCs are generally remarkably aggressive which tend to occur in young women and are associated with large tumor size, poor differentiation, and high rates of node invasion.^2,3^ Moreover, due to a lack of targetable characteristic molecular abnormalities, traditional chemotherapy remains the mainstay of treatment for TNBC.^4^ Unfortunately, early emergence of drug resistance often results in a dismal prognosis for patients with TNBC even if they are highly sensitive to chemotherapy.^5^

To date, anthrayclines- and taxanes-based regimens have been widely used in neoadjuvant and adjuvant chemotherapy for TNBC, but there are no standard regimens to be recommended as the first priority.^6^ As most patients with TNBC have ever been administered with anthracyclines and/or taxanes, it becomes problematic when recurrence or metastasis occurs because other available therapeutic options are limited while the effect of reuse of anthracyclines and taxanes may be unsatisfactory on account of drug resistance. Therefore, studies concerning novel agents to treat mTNBC are warranted, and the results are eagerly awaited.

TNBC is a highly heterogeneous disease, which could be classified into at least 6 subtypes based on gene signatures, the expression of biomarkers, and breast cancer susceptibility gene (BRCA) dysfunction.^5^ Among all the subtypes, the "basal-like breast cancer" accounts for about 85% of TNBC,^1^ whereas the incidence of BRCA mutation varies from 16% to 42%.^7,8^ BRCA-associated breast cancer is closely related to TNBC, and there is an enormous overlap of the biological and clinical characteristics between TNBC and basal-like breast cancer.^2,3^ Both the TNBC/basal-like breast cancer and BRCA-associated breast cancer share a high level of genomic instability, which is possibly due to the aberration of DNA repair mechanisms, thus rationalizing the utilization of DNA-damaging drugs such as platinums and alkylating agents in these patients.^9,10^ In addition, the promising results of platinum-based chemotherapy (PBCT) regimens in the neoadjuvant setting also prompted researchers to further study the effectiveness of platinum as a single agent or in combination therapy for patients with mTNBC after prior exposure to anthracyclines and/or taxanes.^11^

Previous studies have demonstrated the cytotoxic synergy with the combination of platinums and vinorelbine (a semisynthetic vinca alkaloid) in metastatic breast cancer (MBC).^12,13^ However, the exact activity of this regimen in mTNBC has not been fully elucidated. In this article, we reviewed clinical data of patients with mTNBC who failed to anthracyclines and/or taxanes to investigate the potential efficacy of vinorelbine plus platinum (NP) regimen in this subgroup of patients.

## MATERIALS AND METHODS

### Patients

In this study, 41 patients treated with NP regimen for advanced TNBC at Cancer Hospital, Chinese Academy of Medical Sciences between 2001 and 2014 were retrospectively analyzed. All of them have ever received the treatment of anthracyclines and/or taxanes before or after surgery and progressed after a certain period of time.

Data about estrogen receptor, PR, and human epidermal growth factor receptor 2 status were provided by the Pathology Department of Cancer Hospital, Chinese Academy of Medical Sciences. Progress of the disease was diagnosed based on imaging results (X-ray, computed tomography (CT), magnetic resonance imaging, or PET/CT) and/or biopsy of the metastatic lesions. Performance status was recorded according to Karnofsky Performance Status (KPS) Scale. Tumor stage was determined in the light of tumor node metastasis staging system.

This study was a retrospective observational study and patients’ information were collected in the hospital database. There was no direct intervention in patients’ treatment or care. Therefore, ethical approval and a patient's consent are not required.

### Drugs and Treatment

NP combination was delivered in all patients and 4 platinum drugs were used in this study. Among all the platinum-based drugs, cisplatin is one of the original platinum compounds, while carboplatin, lobaplatin, or nedaplatin are the second- or third-generation platinum analogues. One major difference among these platinum drugs is that carboplatin, lobaplatin or nedaplatin could reduce renal, neurological, and gastrointestinal toxicity, which are commonly seen in cisplatin-treated patients. At present, there is no study directly comparing the efficacy of these 4 platinum drugs. In clinical practices, cisplatin and carboplatin are the most commonly used platinum drugs, while lobaplatin and nedaplatin are usually chosen by physicians after taking into account various factors including the patient's age and condition.

In this article, 34 patients received cisplatin (82.9%), 4 patients received carboplatin (9.8%), 2 patients received lobaplatin (4.9%), and 1 patient received nedaplatin (2.4%). Vinorelbine was administered at the dose of 25 mg/m^2^ on days 1 and 8; cisplatin was administered at the dose of 25 mg/m^2^/day from day 1 to day 3 or from day 2 to day 4; Carboplatin was administered at an AUC of 5 dose level on day 2; lobaplatin was administered at the dose of 50 mg/m^2^ on day 2; nedaplatin was administered at the dose of 25 mg/m^2^/day from day 2 to day 4. Treatment cycles were repeated every 3 weeks. The median number of chemotherapy cycles delivered was 3 (range between 2 and 8). One patient continued vinorelbine monotherapy for 3 cycles after the combination therapy. Administration protocols employed in this study are listed in Table 1.

**TABLE 1:**
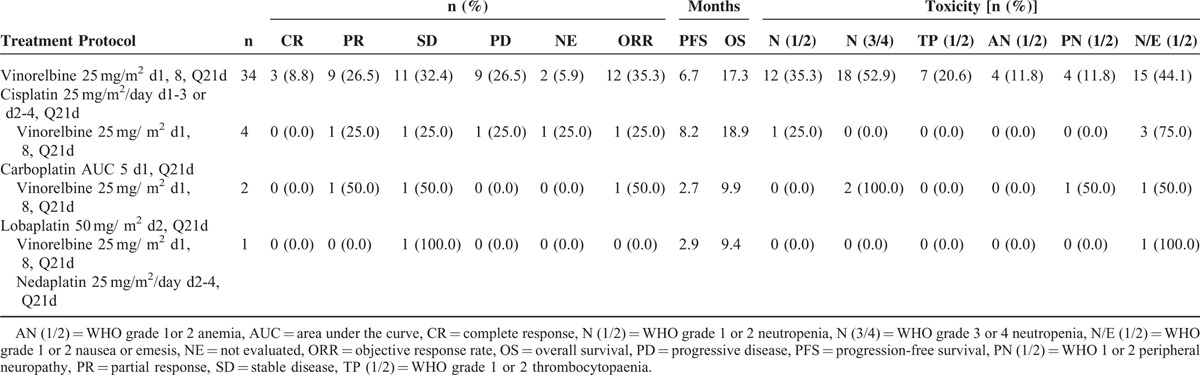
Efficacy and Toxicity of Different NP Protocols

All the patients in this study got the similar daily care.

### Response Evaluation and Follow-up

Tumor response was evaluated by CT scanning every 2 or 3 cycles of chemotherapy during treatment. At follow-up after treatment discontinuation, radiological assessment with CT scanning was conducted every 3 months to monitor changes of the disseminated lesions. Objective response (OR) to chemotherapy was evaluated in strict accordance with RECIST 1.1 guidelines; complete response (CR), partial response (PR), and stable disease (SD) had to maintain 4 weeks after the first confirmation of response by imaging tests.

In this study, the objective response rate (ORR), overall survival (OS), and progression-free survival (PFS) were employed to assess the efficacy of chemotherapy. PFS was defined as the time from initiation of NP combination to disease progression or death from any cause. OS was calculated from the date of first administration of NP regimen to the date of death from any cause or last visit.

### Safety Assessment

Toxicity was evaluated based on reported adverse events and recorded laboratory abnormalities. All the toxicities were classified pursuant to the World Health Organization criteria. Full clinical chemistry workups were performed before every chemotherapy period; whole blood cell count was assessed weekly and before each cycle.

### Statistical Analysis

We used SPSS17.0 program (SPSS Inc., Chicago, IL) to perform the statistical analysis. The means and medians of the variables were calculated by descriptive analysis. Patient characteristics of different subgroups (total responded, partial responded, stable, and progressed) were compared using a χ^2^ test for quantitative data or a Fisher exact probability test for categorical data. Kaplan-Meier method was used for survival analysis and constructing survival curves. Comparison between survival curves is completed using the log-rank test. *P* value less than 0.05 was reckoned as significant for all the analyses.

## RESULTS

### Patient Characteristics

The overall patient characteristics are listed in Table 2. The neoadjuvant and adjuvant treatment history of the patients at initial diagnosis of TNBC are presented in Table 3.

**TABLE 2:**
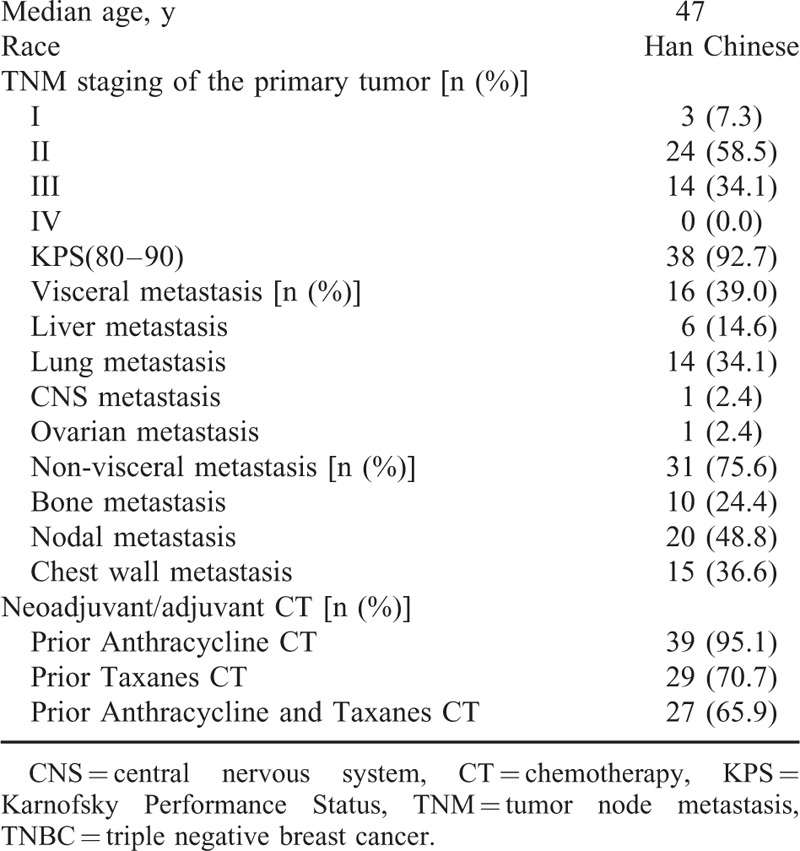
Patient Characteristics

**TABLE 3:**
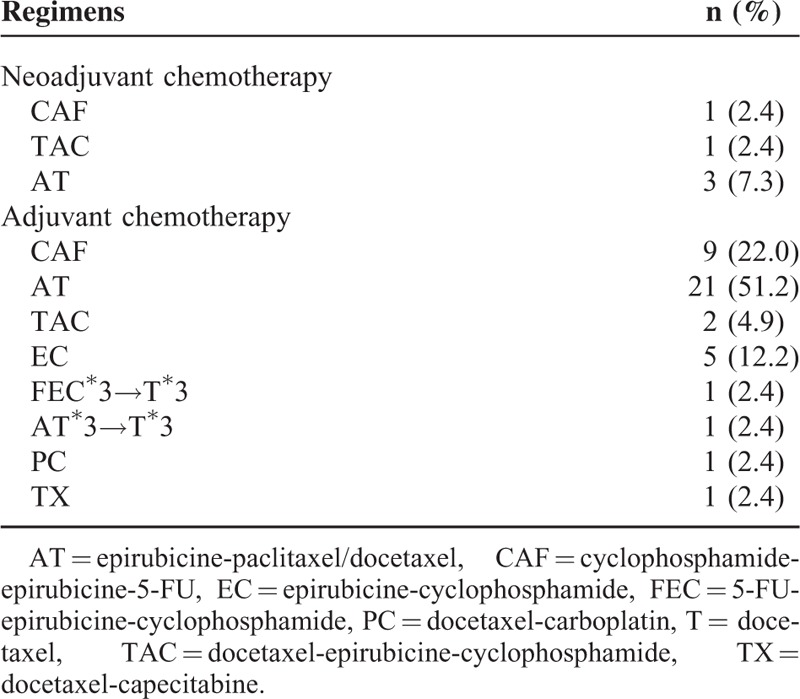
Treatment Protocols of the Patients at Initial Diagnosis

To identify whether special characteristics are related to the efficacy of NP regimen, we further analyzed the separate patient characteristics of different subgroups (total responded, partial responded, stable and progressed). On the whole, no differences were found in patient characteristics among the 4 subgroups. Details were shown in Table 4.

**TABLE 4:**
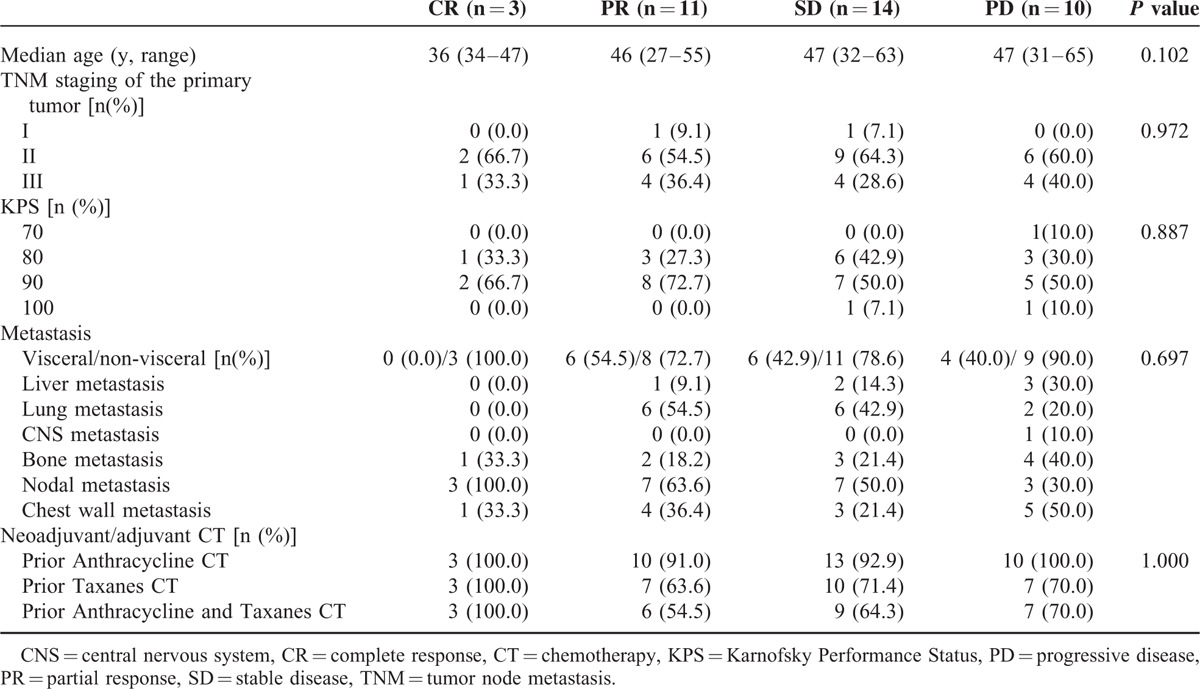
Separate Patient Characteristics of Different Subgroups (Total Responded, Partial Responded, Stable, and Progressed)

### Treatment Outcomes

Objective response (CR + PR) rate was 34.1% (n = 14). Three patients (7.3%) achieved complete response, 11 patients (26.8%) achieved partial response, 14 patients (34.1%) had stable disease, and 10 patients (24.4%) had progressive disease (PD). Efficacy evaluation was not available for 3 patients because the disseminated lesion (2 patients had solitary chest metastasis, 1 patient had ovarian metastasis) was resected before the administration of NP regimen, which was provided as consolidation therapy.

**FIGURE 1:**
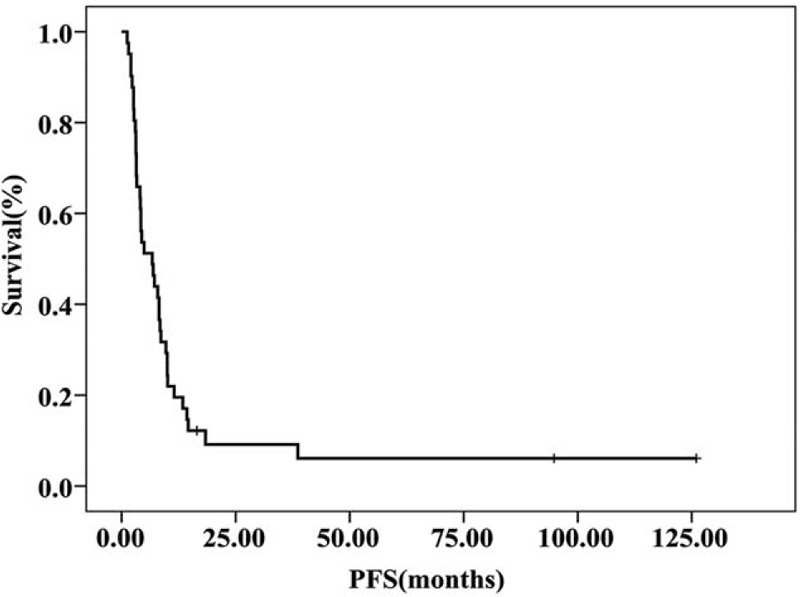
PFS curve for patients with mTNBC treated with NP regimen. The median PFS was 6.7 months (95% CI, 2.9–10.5 months). CI = confidence interval, mTNBC = metastatic triple negative breast cancer, NP = vinorelbine plus platinum, PFS = progression-free survival.

**FIGURE 2:**
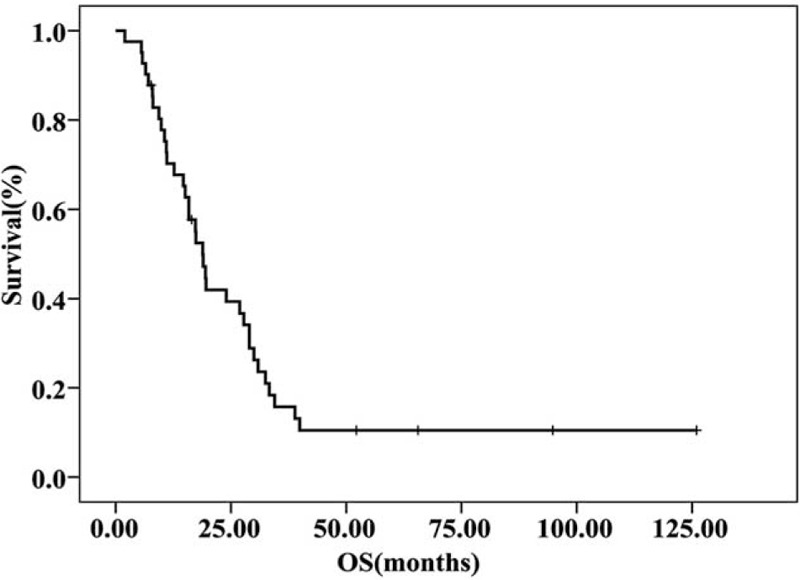
OS curve for patients with mTNBC treated with NP regimen. The median OS was 18.9 months (95% CI, 15.6–22.1 months). CI = confidence interval, mTNBC = metastatic triple negative breast cancer, NP = vinorelbine plus platinum, OS = overall survival.

**FIGURE 3:**
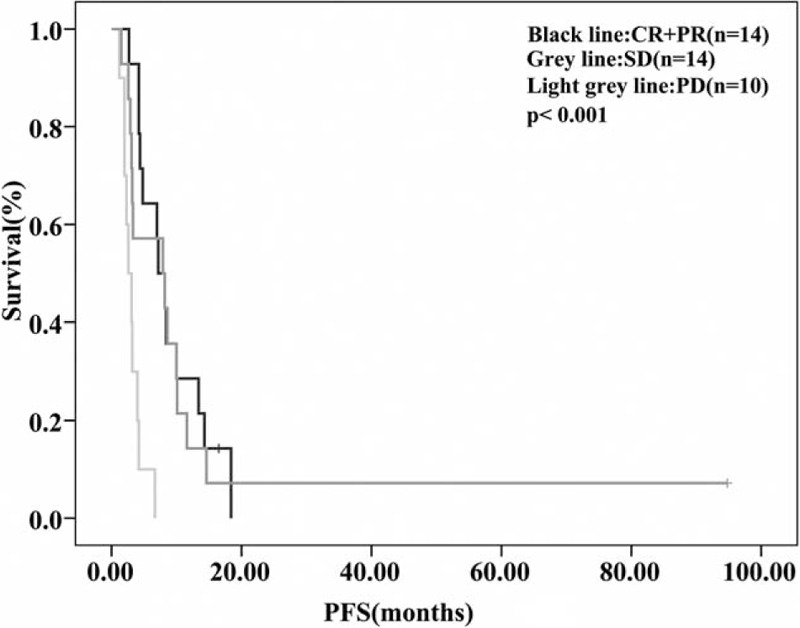
PFS according to response to NP regimen. The median PFS was 7.2 months (95% CI, 4.9–9.5 months) for patients who responded to NP regimen, 7.9 months (95% CI, 0.0–16.9 months) for patients with stable disease, and 2.6 months (95% CI, 1.4–3.8 months) for patients who had PD (*P *< 0.001). CI = confidence interval, NP = vinorelbine plus platinum, OS = overall survival, PD = progressive disease, PFS = progression-free survival.

**FIGURE 4:**
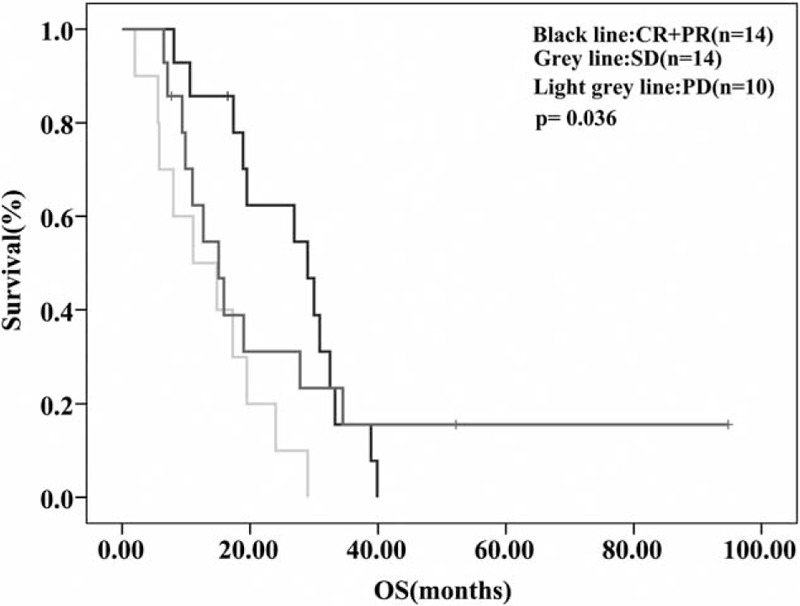
OS according to response to NP regimen. The median OS was 29.0 months (95% CI, 16.8–41.2 months) for patients who responded to NP regimen, 15.1 months (95% CI, 9.5–20.8 months) for patients with stable disease, and 11.1 months (95% CI, 0.8–21.5 months) for patients who had PD (*P* = 0.036). CI, confidence interval, NP = vinorelbine plus platinum, OS = overall survival, PD= progressive disease.

The median follow-up time was 36.8 months. For the overall population (n = 41), median PFS and median OS were 6.7 months (95% confidence interval (CI), 2.9–10.5 months) and 18.9 months (95% CI, 15.6–22.1 months), respectively (Fig. 1 and Fig. 2). In the additional analysis, we found median PFS and OS were improved in patients who responded to NP regimen (CR+PR) or had stable disease: PFS for patients with CR/PR, SD, and PD were 7.2 months (95% CI, 4.9–9.5 months), 7.9 months (95% CI, 0.0–16.9 months), and 2.6 months (95% CI, 1.4–3.8 months), respectively (*P *< *0.001)*; OS for patients with CR/PR, SD, and PD were 29.0 months (95% CI, 16.8–41.2 months), 15.1 months (95% CI, 9.5–20.8 months), and 11.1 months (95% CI, 0.8–21.5 months), respectively (*P* = 0.036), as shown in Figure 3 and Figure 4.

As 4 platinum drugs were used in combination with vinorelbine to treat patients with mTNBC in this report, we also presented the different efficacy and toxicity of these 4 NP regimens in Table 1. However, 34 of 41 patients received the treatment of vinorelbine-cisplatin regimen, while only 4 patients were administered with vinorelbine-carboplatin, 2 patients with vinorelbine-lobaplatin, and 1 patient was treated with vinorelbine-nedaplatin. Therefore, direct comparison of the efficacy or toxicity among the 4 NP regimens was not possible.

### Tolerance

The most frequently reported hematologic adverse event was neutropenia (n = 33, 80.5%); grade 3/4 neutropenia occurred in 20 patients (48.8%), and they all received the treatment of granulocyte colony-stimulating factor. There was no febrile neutropenia observed in this study. In addition, thrombocytopenia was noted in 8 patients (19.5%) and anemia was reported in 5 patients (12.2%). Nausea and emesis were the most notable non-hematologic adverse events. Twenty patients (48.8%) had mild nausea and emesis (grade 1/2), and grade 3 nausea/emesis was observed in 5 patients (12.2%). Grade 1/2 peripheral neuropathy occurred in 12.2% of all cases (5 patients). Impaired renal function was recorded in 1 patient (2.4%). Chemical phlebitis was only observed in 1 patient, the incidence of which was very low (2.4%). One patient (2.4%) experienced hoarseness from the use of cisplatin.

Together, no treatment-related death was observed. Two patients required discontinuation of chemotherapy because of the comorbidity of upper gastrointestinal hemorrhage and grade 4 anemia, respectively. One patient had dose reduction because of grade 2 neutropenia. Delaying the administration of vinorelbine for grade 2 vomiting and grade 3 neutropenia was noted in 1 patient.

## DISCUSSION

In recent years, TNBC has intrigued great interest among researchers not only because of its aggressive pathological and clinical features but also its easily acquired resistance to commonly used chemotherapy which makes the subsequent treatment after tumor progression very intractable.^2,3,13–16^ In order to optimize treatment strategies and ultimately improve the prognosis of patients with TNBC, great efforts have been made in understanding the potential therapeutic targets of TNBC. However, chemotherapy combined with targeted agents such as poly ADP-ribose polymerase inhibitor iniparib, epidermal growth factor receptor inhibitor cetuximab, and anti-angiogenic agent bevacizumab has unfortunately failed to improve the outcomes of patients with TNBC significantly.^17^ Hence, cytotoxic chemotherapy still remains the mainstay treatment for patients with TNBC. Nevertheless, validated data from phase III trials on the chemotherapy of TNBC are absent, and most comparisons between chemotherapy regimens are retrospective in nature. Therefore, there are no recommendations that could be proposed for patients with TNBC, and treatment for TNBC should be selected as it is for other breast-cancer subtypes. Both anthracyclines and taxanes are classic therapeutic agents for breast cancer, which are extensively utilized in treating all subtypes of breast cancer including TNBC.^6^ In fact, most patients with TNBC were anthracyclines- and taxanes-pretreated, while treating patients who failed to these 2 agents is usually difficult because medication history decreases the number of treatment options for metastatic disease.

For advanced breast cancer, treatment options include agents such as platinums, capecitabine, vinorelbine, gemcitabine, ixabepilone, and their combinations,^18–21^ which could also be considered as reasonable choices for mTNBC. It should be noted that in recent years, there has been a renewed interest in platinums for TNBC treatment in the metastatic setting. Their use is supported by the fact that there is a high frequency of germline mutations in BRCA1/2 gene in TNBC. For sporadic patients with TNBC, who are not carriers of BRCA1 mutation, there is also evidence showing BRCA1 pathway dysfunction which is probably related to the epigenetic mechanisms.^22^ It has been established that BRCA1/2 are essential for maintaining genomic stability by involving in DNA-damage repair, and dysfunction of BRCA1/2 and their pathways can result in a specific DNA-repair defect which will sensitize patients with TNBC to interstrand cross-linking agents such as platinums.^23^ In addition, Leong CO et al reported that the p53 family member p63 could control a survival pathway for p73-dependent cisplatin sensitivity specific to TNBC.^24^

According to the above theoretical basis, some studies have been conducted to explore the effects of PBCT in the neoadjuvant setting for TNBC. In 2006 SABCS meeting, Garber first reported that cisplatin monotherapy can produce an ORR of 50% and a 22% pathological complete remission in patients with TNBC.^25^ This was followed by a series of similar reports. In 2014, using meta-analysis, Lonati V et al demonstrated that the pathological complete remission rates could be increased signiﬁcantly with the addition of cisplatin or carboplatin in TNBC compared with neoadjuvant chemotherapy containing no platinum drugs.^11^

These promising results have promoted further investigation of the use of platinums for TNBC in the adjuvant and metastatic settings. Two clinical trials showed that platinum as a single agent has a poor efficacy in mTNBC.^26,27^ Therefore, platinum-based combination regimens merit further studies for their potential in treating TNBC with highly aggressive biological behaviors. Recently, a phase II study concerning the effects of gemcitabine plus cisplatin (GP) combination as the first-line therapy in mTNBC reported that the ORR was 62.5%, while the PFS and OS were 7.2 months (95% CI, 5.6–8.9 months) and 19.1 months (95% CI, 12.4–25.8 months), respectively.^10^ Fan et al reported that the docetaxel-cisplatin (TP) regimen might be superior to the docetaxel-capecitabine (TX) regimen as the first-line therapy for patients with mTNBC whoever received anthracyclines or taxanes. The ORR of the docetaxel-cisplatin group was higher than that of the docetaxel-capecitabine group (63.0% vs 15.4%, *P* = 0.001) and both the PFS and OS improved significantly (10.9 vs 4.8 months, *P* < 0.001; 32.8 vs 21.5 months, *P* = 0.027).^28^ Staudacher L et al reported that among 143 patients treated for MBC with PBCT, no difference in OS or PFS was observed between patients with TNBC and patients without TNBC, despite the fact that TNBC is known to have a poorer overall prognosis. Therefore, the authors assumed that PBCT could improve the spontaneous poor prognosis of metastatic TNBC^9^.

Doubtlessly, we could conclude from the above results that PBCT are of great potential in treating patients with mTNBC. But the question is which chemotherapeutic agent could be combined with platinums in mTNBC. In fact, docetaxel, gemcitabine, and vinorelbine are all common choices for treating patients with mTNBC. There have been clinical trials evaluating the effects of gemcitabine plus cisplatin and docetaxel-cisplatin regimen in mTNBC, but data about the NP regimen in patients with mTNBC are absent, which is our focus in this study.^10,28^ Vinorelbine is a semisynthetic vinca alkaloid that emerges as one of the most active drugs in breast cancer. Its single-agent activity in the first- and second-line treatment of MBC is relatively high with response rates of 41% to 50% and 30%, respectively.^29^ Vassilomanolakis et al^29^ has already demonstrated the efficacy of NP regimen in patients with MBC previously treated with anthracyclines: the ORR was 49% (95% CI, 35%–63%), and the median time to progression was 5 months and median OS 12 months. Another study assessing the activity of NP combination as a salvage regimen in patients with MBC previously treated with anthracyclines and taxanes showed that the ORR was 47.2% (95% CI, 31%–63%), time to progression was 16 weeks, and OS was 36 weeks.^13^ In our study, the ORR was 34.1%, PFS was 6.7 months, and OS 18.9 months, which suggests great potential of NP regimen in mTNBC pretreated with anthracyclines and taxanes. It seems that both the PFS and OS in patients with mTNBC were longer than that of the general MBC patients in previous studies.^13^ However, whether NP regimen is specific to patients with TNBC needs to be explored with further clinical trials.

In the subgroup analysis, we found that compared with patients who had progressed disease, patients who responded to NP regimen (CR+PR) or had stable disease had significantly improved PFS and OS (*P* < 0.001). The median PFS was 7.2 months (95% CI, 4.9–9.5 months) for patients who achieved CR or PR, 7.9 months (95% CI, 0–16.9 months) for patients with stable disease, and 2.6 months (95% CI, 1.4–3.8 months) for patients who had PD (Fig. 3). The median OS was 29.0 months (95% CI, 16.8–41.2 months), 15.1 months (95% CI, 9.5–20.8 months), and 11.1 months (95% CI, 0.8–21.5 months) respectively (*P* = 0.036), as shown in Figure 4. Hence, clinical complete, partial remission or stable disease is the ideal result that we eagerly pursue. But even classified into one subgroup (TNBC), patients could have different responses to the same chemotherapy regimen. Reasons for this phenomenon could be very complicated, which may involve different patient characteristics, biological diversity within TNBC, the intrinsic or acquired resistance of cancer cells and some undefined molecular mechanisms. Nevertheless, researches regarding this area are lacking, and elucidating this question requires further analyses.

In this study, we provided a primary analysis of the separate patient characteristics of different subgroups (total responded, partial responded, stable, and progressed) to demonstrate whether different patient characteristics could affect the treatment responses (see Table 4). Our results indicated no differences in patient characteristics among the 4 subgroups. However, as the sample size is small, we cannot yet conclude that patient characteristics have no influences on the efficacy of NP regimen in the treatment of patients with mTNBC. More efforts are needed to identify which group of patients with TNBC could be more sensitive to NP regimen, which may help optimize clinical choices and improve patients’ prognosis.

As NP regimen has been well studied and reported, adverse events were not the main focus of our study. Hematologic toxicity was the most commonly seen adverse event: grade 3/4 neutropenia occurred in 20 patients (48.8%); there was no neutropenia febrile. The main nonhematologic toxicity was grade 1/2 nausea and emesis (n = 20, 48.8%). All the toxicities were manageable.

Although 4 platinum drugs were used in this study, the number of patients who received carboplatin, lobaplatin, or nedaplatin was too small for any detailed or credible statistical analysis, hence making it impossible to identify which treatment combination had better efficacy. Because carboplatin, lobaplatin, or nedaplatin could vastly reduce the side effects, physicians could replace cisplatin with these second- or third-generation platinum drugs when patients had more unfavorable features.

In conclusion, NP regimen showed clinical activity in patients with mTNBC and the toxicity was acceptable and manageable. However, the caveat is that this study was retrospective in nature and patients were not randomized. Besides, the small sample size is also a limitation of this study. TNBC is a heterogeneous disease, responses to NP combination may differ among distinct subtypes of TNBC. Despite the limitations, the promising results of this study still support further trials to validate the efficacy of NP regimen in mTNBC pretreated with anthracyclines and/or taxanes.
